# Microstructures and Electrical Conduction Behaviors of Gd/Cr Codoped Bi_3_TiNbO_9_ Aurivillius Phase Ceramic

**DOI:** 10.3390/ma14195598

**Published:** 2021-09-26

**Authors:** Huajiang Zhou, Shaozhao Wang, Daowen Wu, Qiang Chen, Yu Chen

**Affiliations:** 1 School of Mechanical Engineering, Chengdu University, Chengdu 610106, China; zhouhj1996@163.com (H.Z.); wangsz0908@163.com (S.W.); wdw132812976202021@163.com (D.W.); 2 College of Materials Science and Engineering, Sichuan University, Chengdu 610065, China; cqscu@scu.edu.cn

**Keywords:** Aurivillius phase ceramic, Bi_3_TiNbO_9_, electrical conduction behaviors, impedance spectrum, electrical modulus

## Abstract

In this work, a kind of Gd/Cr codoped Bi_3_TiNbO_9_ Aurivillius phase ceramic with the formula of Bi_2.8_Gd_0.2_TiNbO_9_ + 0.2 wt% Cr_2_O_3_ (abbreviated as BGTN−0.2Cr) was prepared by a conventional solid-state reaction route. Microstructures and electrical conduction behaviors of the ceramic were investigated. XRD and SEM detection found that the BGTN−0.2Cr ceramic was crystallized in a pure Bi_3_TiNbO_9_ phase and composed of plate-like grains. A uniform element distribution involving Bi, Gd, Ti, Nb, Cr, and O was identified in the ceramic by EDS. Because of the frequency dependence of the conductivity between 300 and 650 °C, the electrical conduction mechanisms of the BGTN−0.2Cr ceramic were attributed to the jump of the charge carriers. Based on the correlated barrier hopping (CBH) model, the maximum barrier height *W_M_*, dc conduction activation energy *E_c_*, and hopping conduction activation energy *E_p_* were calculated with values of 0.63 eV, 1.09 eV, and 0.73 eV, respectively. Impedance spectrum analysis revealed that the contribution of grains to the conductance increased with rise in temperature; at high temperatures, the conductance behavior of grains deviated from the Debye relaxation model more than that of grain boundaries. Calculation of electrical modulus further suggested that the degree of interaction between charge carriers *β* tended to grow larger with rising temperature. In view of the approximate relaxation activation energy (~1 eV) calculated from *Z″* and *M*″ peaks, the dielectric relaxation process of the BGTN−0.2Cr ceramic was suggested to be dominated by the thermally activated motion of oxygen vacancies as defect charge carriers. Finally, a high piezoelectricity of *d*_33_ = 18 pC/N as well as a high resistivity of *ρ*_*dc*_ = 1.52 × 10^5^ Ω cm at 600 °C provided the BGTN−0.2Cr ceramic with promising applications in the piezoelectric sensors with operating temperature above 600 °C.

## 1. Introduction

Bismuth layer—structured ferroelectric (BLSF) compounds (also called Aurivillius phase compounds), with the general formula [Bi_2_O_2_][A*_m_*_−1_B*_m_*O_3*m*+1_], where A with a dodecahedral coordination is a low valence element (less than or equal to trivalent), B with a octahedral coordination is a transition metal element (e.g., Cr^3+^, Ce^4+^, Ti^4+^, Nb^5+^, Ta^5+^, W^6+^), and 1 ≤ *m* ≤ 6, constructed by *m*[ABO_3_]^2−^ layers that alternate with [Bi_2_O_2_]^2+^ layers [1,2,3]. Due to their relatively high Curie point (*T*_C_) and excellent fatigue resistance, they have attracted extensive attention for their potential application in high-temperature piezoelectric systems [4,5] and ferroelectric random-access memory (FeRAM) [6]. However, the piezoelectricity of these compounds is limited because of the 2D orientation restriction of their spontaneous polarization (*P*_s_) rotation and their high coercive fields (*E*_c_), and high conductivity also restricts their applications in high temperature environment [7]. The conductivity mechanisms of most ferroelectric materials are approximately divided into three categories: electronic conduction, oxygen vacancies ionic conduction, and mixed conduction of ions and holes. BLSF compounds’ conduction mechanisms are still indistinct. Various mechanisms have been put forward to explain the conductivity property. These mechanisms may be very correct according to the corresponding experiments, but none of them is widely accepted.

Bi_3_TiNbO_9_ (BTN), which is made up of (Bi_2_O_2_)^2+^ layers between which two (BiTiNbO_7_)^2−^ layers (*m* = 2) are inserted, has a very high *T*_C_ of ~914 °C. As a sensing material, BTN is promising for fabricating piezoelectric accelerometers with operating temperature above 500 °C [8], which can be used for the high-temperature vibration monitoring of some large power equipment such as aircraft engines, gas turbines, power generators, etc. However, the piezoactivity of pure BTN ceramics is very low (*d*_33_ ≤ 7 pC/N) [9]. The resistivity of BTN is only about 10^7^ Ω·cm at 400 °C, for example [10]. Up to now, most reported studies about BTN have concentrated on its crystal structure [11,12,13,14], the electrical properties of pure BTN ceramics [15,16,17,18], and improvement in its piezoelectric ability [19,20,21]. For example, S.V. Zubkov doped Gd elements in BTN, which can increase the Curie temperature to 950 °C [22]. Gd element was also found to provide high insulation and low loss [23]. Chen et al. codoped W/Cr into Bi_4_Ti_3_O_12_ of BLSFs, which increased the resistivity (*σ*_*dc*_ (600 °C)) and piezoelectric properties (*d*_33_ (RT)) to 2.94 × 10^6^ Ω·cm and 28 pC/N, respectively [24,25]. In addition, Chen et al. codoped Mo/Cr into CaBi_2_Nb_2_O_9_, which increased the multifaceted performances of CaBi_2_Nb_2_O_9_ (*d*_33_ = 15 pC/N, *T*_C_ = 939 °C, *σ*_*dc*_ (600 °C) = 3.33 × 10^5^ Ω·cm) [26]. However, there has been no significant improvement of the piezoelectric properties of BTN-based ceramics. There is also no clear understanding of their conduction behavior.

In this work, we studied the effects of Gd/Cr codoped on the microstructure, AC conduction mechanisms, and electrical impedance spectrum of BGTN−0.2Cr ceramic, focusing on the effect of grain size on conduction mechanism and impedance spectroscopy. A type of piezoelectric ceramic that can be used as a sensing material for piezoelectric sensors with operating temperatures above 600 °C was developed in this work.

## 2. Experimental Section

### 2.1. Sample Preparation

A kind of Gd/Cr codoped Bi_3_TiNbO_9_ Aurivillius phase ceramics, with the formula of Bi_2.8_Gd_0.2_TiNbO_9_ + 0.2 wt% Cr_2_O_3_ (abbreviated as the BGTN−0.2Cr ceramic hereafter), was prepared by using the conventional solid-state reaction route in two steps. First, the metal oxides Bi_2_O_3_ of 99% purity, Gd_2_O_3_ of 99.99% purity, TiO_2_ of 99% purity, and Nb_2_O_5_ of 99.99% purity (as raw materials) were weighed according to the stoichiometric ratio (Bi_2_O_3_, Gd_2_O_3_, TiO_2_ and Nb_2_O_5_ produced in Chron Chemicals, Chengdu, China; Cr_2_O_3_ produced in Aladdin, Shanghai, China). These raw materials were mixed evenly by ball milling for 6 h, using alcohol as solvent and zirconia balls as grinding media. The dried mixture was calcined at 850 °C for 4 h to synthesize the components of Bi_2.8_Gd_0.2_TiNbO_9_. Second, the calcined powders, with *x* wt% Cr_2_O_3_ of 99.95% purity added, were ground for 12 h under the same grinding conditions and granulated with polyvinyl alcohol (abbreviated as PVA, produced in Chron Chemicals, Chengdu, China) as a binder. The powders were pressed into discs with a diameter of 10 mm and a thickness of 0.8 mm under an isostatic pressure of 10 MPa. After firing at 650 °C for 2 h to burn out the PVA, these discs were sintered in a sealed alumina crucible at 1100 °C for 2 h to obtain the dense ceramics. Finally, Ag electrodes were screen-printed on both surfaces of the polished ceramics and fired at 700 °C for 10 min. For comparison, the pure Bi_3_TiNbO_9_ ceramic was prepared by the same process.

### 2.2. Sample Characterization

The crystallographic structure of the samples was characterized by X-ray diffractometer (XRD, DX−2700B, HAOYUAN INSTRUMENT, Dandong, China) using CuK_α_ radiation. The microscopic morphology of the samples was observed by scanning electron microscope (SEM, Quanta FEG 250, FEI, Waltham, MA, USA). The elemental analysis of the samples was carried out by the energy-dispersive X-ray spectroscopy (EDS) attached to the scanning electron microscope. In the frequency range of 100 Hz–100 kHz, the dielectric constant and loss of the samples as a function of temperature (room temperature~650 °C) were detected by an LCR meter (TH2829A, Tonghui Electronic, Changzhou, China) attached to a programmable furnace. At various temperatures (room temperature~650 °C), the AC impedance spectrum of the samples as a function of frequency (20 Hz–2 MHz) was measured by using an impedance analyzer (TH2838, Tonghui Electronic) attached to a programmable furnace. The electromechanical resonance spectroscopy of the poled samples was investigated by a broadband LCR digital bridge in the frequency range from 280 to 390 kHz. The planar electromechanical coupling factors *k*_p_, mechanical quality factors *Q*_m_, and planar frequency constant *N*_p_ were determined according to Onoe’s equations [27]. The piezoelectric charge coefficient of the samples was measured using a quasistatic *d*_33_/*d*_31_ m (ZJ−6AN, IACAS, Beijing, China).

## 3. Results and Discussion

### 3.1. Phase Structure of Ceramics

**Figure 1:**
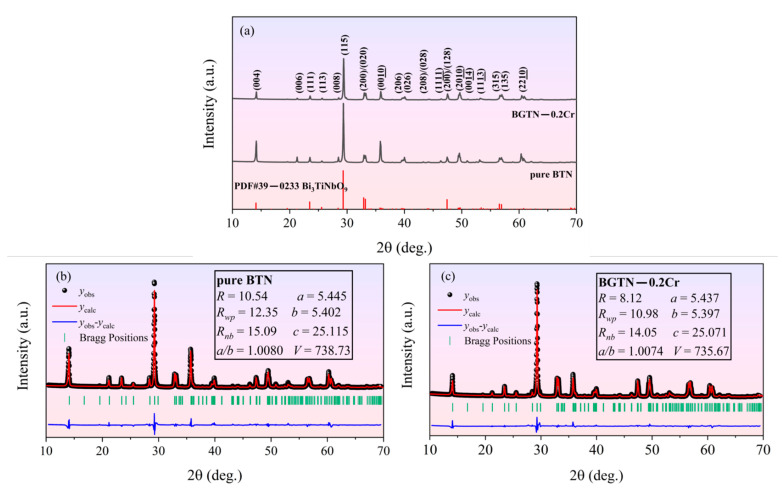
XRD analysis of the sintered ceramics: (**a**) observed XRD patterns of the pure BTN ceramic and the BGTN−0.2Cr ceramic; (**b**) Rietveld XRD refinement of the pure BTN ceramic; (**c**) Rietveld XRD refinement of the BGTN−0.2Cr ceramic.

Figure 1 shows the XRD analysis of the sintered ceramics. As can be seen from Figure 1a, both for the pure BTN ceramic and the BGTN−0.2Cr ceramic, their observed XRD patterns agree with the standard X-ray diffraction powder patterns from JCPDS card No. 39-0233 well. Therefore, both samples were identified as the pure Bi_3_TiNbO_9_ phase with orthorhombic structure and space group of *A21am*. No second phase was found in the XRD pattern of the BGTN−0.2Cr ceramic. The introduced Gd_2_O_3_ and Cr_2_O_3_ formed a complete solid solution with Bi_3_TiNbO_9_. The strongest diffraction peak of the BGTN−0.2Cr ceramic was the (1 × 1 × 5) peak, which is consistent with the rule of the strongest diffraction peak of BLSF ceramics (1 × 1 × 2m + 1) [28]. In view of the similar ionic radius, it is believed that Gd^3+^ (*r* = 0.0938 nm) entered the A-site (Bi^3+^: *r* = 0.103 nm) of the perovskite unit while Cr^3+^ (*r* = 0.0615 nm) entered the B-site (Ti^4+^: *r* = 0.0605 nm). Meanwhile, the stability of the ABO_3_—type perovskite structure can be described by the tolerance factor (*t*), which can be expressed as [29]:

(1) $$t=\frac{r_{A}+r_{o}}{\sqrt{2}(r_{B}+r_{o})}$$

where *r_A_*, *r_B_*, and *r_o_* are the ionic radii of A, B, and the oxygen ion, respectively. The perovskite structure remains stable when *t* is between 0.77 and 1.10. However, the value of the tolerance factor may be further limited in BLSF due to the structural incompatibility between the pseudo perovskite layer and the bismuth-oxygen layer, which is caused by the mismatching of their transverse dimension. Subbarao [1] proposed that when *m* = 2, *t* is limited in the range of 0.81~0.93. We obtained *t* = 0.86 for BTN and *t* = 0.82 for BGTN−0.2Cr. The decrease in the tolerance factor could demonstrate the successful substitution of Gd^3+^ and Cr^3+^ for Bi^3+^ and Ti^4+^ at the A- and B-sites, respectively, in the perovskite unit of BTN.

The Rietveld refinement was performed for the XRD patterns of the pure BTN ceramic and the BGTN−0.2Cr; the refined factors and cell parameters are shown in Figure 1b,c. When compared with pure BTN, BGTN−0.2Cr showed a contracted unit cell as well as a smaller orthorhombic distortion (a/b). The substitution of Gd^3+^ for Bi^3+^ at the A—site tended to induce a principle change in the structural distortion of perovskite blocks composed of (Ti, Nb)O_6_ octahedrons. However, the substitution of Cr^3+^ for Ti^4+^ at the B—site may have decreased the tilting angle of the (Ti, Nb)O_6_ octahedron, leading to reduced distortion in the perovskite blocks.

### 3.2. Grain Morphology and Chemical Composition of Ceramics

**Figure 2:**
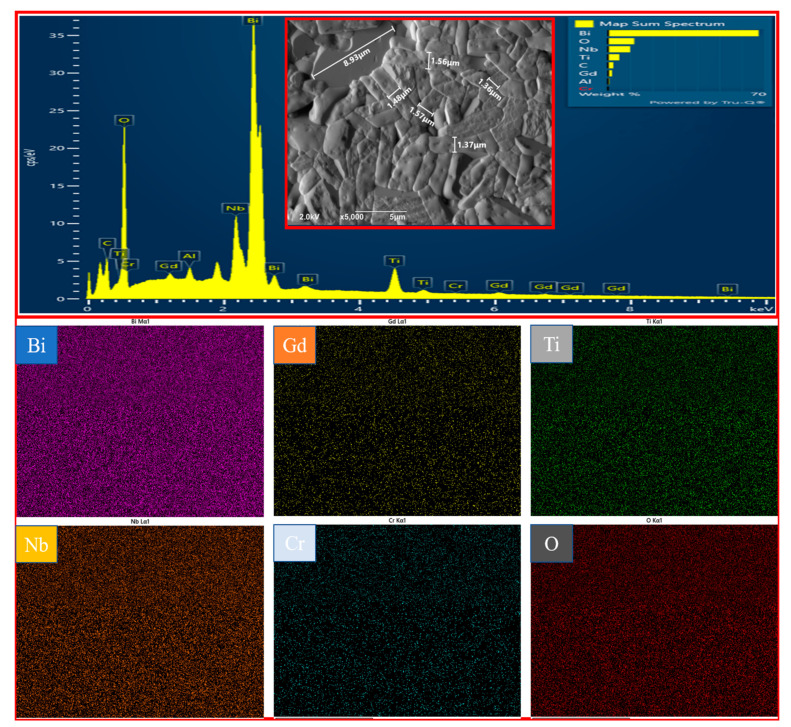
SEM and EDS analysis of the BGTN−0.2Cr ceramic, focused on its thermal-etched surface (the grain size was determined using the intercept procedure on the basis of the SEM image).

Figure 2 shows the SEM and EDS analysis of the BGTN−0.2Cr ceramic, focused on its thermal-etched surface. As can be seen from the SEM image inserted in the EDS, a dense microstructure with well-defined grain boundaries was formed in the ceramic. All the grains were closely stacked, with random orientation, which agrees with the weaker intensity of the (0 × 0 × *l*) diffracted peaks observed in Figure 1. The microstructure was composed of plate−like grains. Such grain growth possessed a high anisotropy such that the length (*L*) was larger than the thickness (*T*), which can be attributed to the higher grain growth rate in the direction perpendicular to the *c*-axis of the BLSF grains [30]. It is well known that the crystal grain aspect ratio (*L/T*) has a significant influence on the resistivity of BLSF ceramics. A higher aspect ratio is often related to higher resistivity [31]. The average thickness of these plate-like grains was 1.47 μm, while the length was about 8.93 μm. The high aspect ratio, with a *L/T* value of 6.1, was expected to lend higher resistivity to the BGTN−0.2Cr ceramic. Furthermore, EDS analysis showed that the BGTN−0.2Cr ceramic contained six elements: Bi, Gd, Ti, Nb, Cr, and O. All the elements presented a uniform distribution in the detected zone. Both gadolinium and chromium were successfully incorporated into the BTN.

### 3.3. Electrical Conduction Behaviors of Ceramics

**Figure 3:**
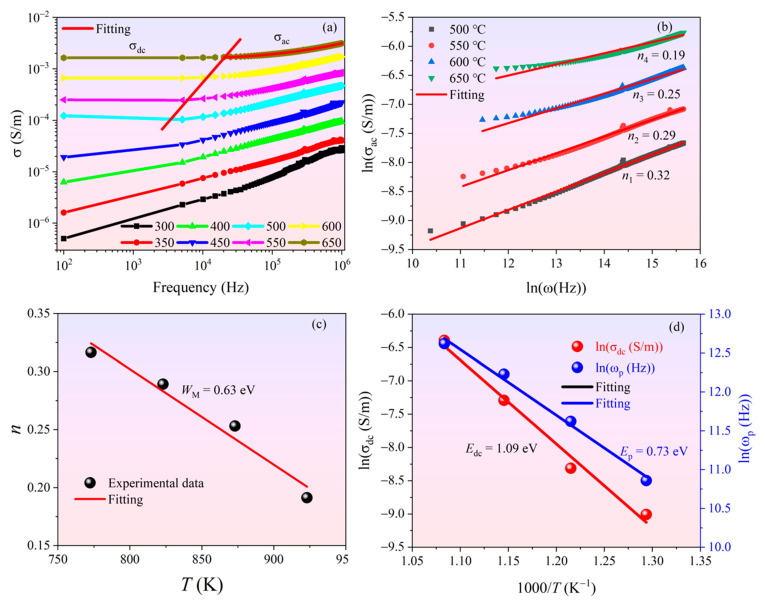
Electrical conduction spectroscopy of the BGTN−0.2Cr ceramic at high temperature: (**a**) frequency dependence of the total conductivity; (**b**) relationship between ac conductivity and angular frequency; (**c**) values of the frequency index factor *n* calculated by the general Jonscher’s theory; (**d**) Arrhenius fitting of the plots of lnσ_dc_ and lnω_*p*_ vs. 1000/*T*.

In order to further understand the conduction mechanism at high temperature, the results of electrical conduction spectroscopy of the BGTN−0.2Cr ceramic at high temperature are shown in Figure 3. It can be seen from Figure 3a that the conductivity of the BGTN−0.2Cr ceramic did not change with frequency in the low−frequency section at various temperatures, while in the high-frequency section, the conductivity increased with the increase in frequency. This is consistent with the jump relaxation model proposed by Funke [32]. In the low-frequency section, the migration of charge carriers was mainly implemented through long-distance jump, which led to direct current conductivity. As the frequency increased, the mobility of charge carriers was gradually limited, and the conductivity became positively related to frequency. The functional relationship between conductivity and frequency is consistent with the general Jonscher’s theory [33]:

(2) $$\sigma \left(\omega \right)=\sigma_{dc}\left(T\right)+A\omega^{n}\;\left(n=0\sim 1\right)$$

where σ(ω) is the total conductivity, $\sigma_{dc}\left(T\right)$ is the dc conductivity, *A* is a constant with temperature dependence, ω is the angular frequency, *n* is the frequency index factor, and $A\omega^{n}$ represents the ac conductivity. $\sigma_{dc}\left(T\right)$, *n*, and *A* can be fitted by Equation (2). Based on Jonscher’s theory, the frequency dependence of ac conductivity originated from the relaxation of the ionic atmosphere after the movement of charge carriers, as shown in Figure 3a.

The changes in the temperature-related frequency index factor *n* provide information for the origins of conductance. The value of *n* in the correlated barrier−hopping (CBH) model decreases with temperature rising [34]. The results obtained for the BGTN−0.2Cr ceramic are shown in Figure 3b,c and are well in line with the CBH model. The first-order approximation of the frequency index factor *n* of the CBH model is given as Equation (3) [35]:

(3) $$n=1-\frac{6K_{B}T}{W_{M}}$$

where *W_M_* is the maximum barrier height. The calculated *W_M_* (~0.63 eV) was slightly less than the oxygen vacancy’s activation energy (~0.6–1.2 eV), indicating that oxygen vacancies were the main charge carriers between local states. This may be related to the segmental ionization of the first- and second-order oxygen vacancies, which can be expressed by Equations (4) and (5), respectively:

(4) $$V_{O}↔V_{O}^{.}+e^{‘}$$

(5) $$V_{O}^{.}↔V_{O}^{¨}+e^{‘}$$

where $V_{O}^{.}$ and $V_{O}^{¨}$ are the first- and second-stage ionized oxygen vacancies, respectively. In many BLSF materials, the activation energies of the first- and second-stage ionized oxygen vacancies (E_I_ and E_II_) have been reported as ~0.5 eV and ~1.2 eV, respectively [36]. For instance, E_I_ and E_II_ in SBTW0.04 are 0.57 eV and 0.74 eV [37], and E_II_ in Bi_2.8_Nd_0.2_NbTiO_9_ is 0.9 eV [38]. The conductive and relaxation behaviors related to these BLSF materials can be attributed to long-range/local migration of two-stage ionized oxygen vacancies.

The conductivity of ion conductive materials is related not only to movable ion concentrations but also to ion jump frequencies [39]. The hopping angular frequency ω_*p*_ of ac conductivity can be fitted by Equation (6), and the activation energy of dc conduction and hopping conduction can be determined and calculated by Arrhenius fitting shown in Equations (7) and (8):

(6) $$\omega_{p}={\left(\frac{\sigma_{ac}}{A}\right)}^{1/n}$$

(7) $$\omega_{p}=\omega_{0}\exp\left(-E_{p}/kT\right)$$

(8) $$\sigma_{dc}=\sigma_{0}\exp\left(-E_{dc}/kT\right)$$

where *ω*_0_ and *σ*_0_ are pre−exponential factors, *k* is the Boltzmann constant, *E_p_* is the activation energy of hopping conduction, and *E_dc_* is the dc conduction activation energy. The calculated *E_dc_* (~1.09 eV) in Figure 3d was slightly smaller than the activation energy (~1.2 eV) of the second ionized oxygen vacancies, which indicates that $V_{O}^{.}$ and $V_{O}^{¨}$ were involved in conduction during the dc conductance process. Furthermore, the reduction of *E_p_* (0.73 eV) indicates that when the oxygen vacancies migrated from long- to short-range jumps, the activation energy decreased, which may have increased the carrier mobility during the ac conduction process. *E_p_* was slightly greater than *W_M_*, indicating that carriers were over the barrier height and then had a short-distance jump participation in the ac conduction process. The higher *E_p_* may be also caused by relaxation.

**Figure 4:**
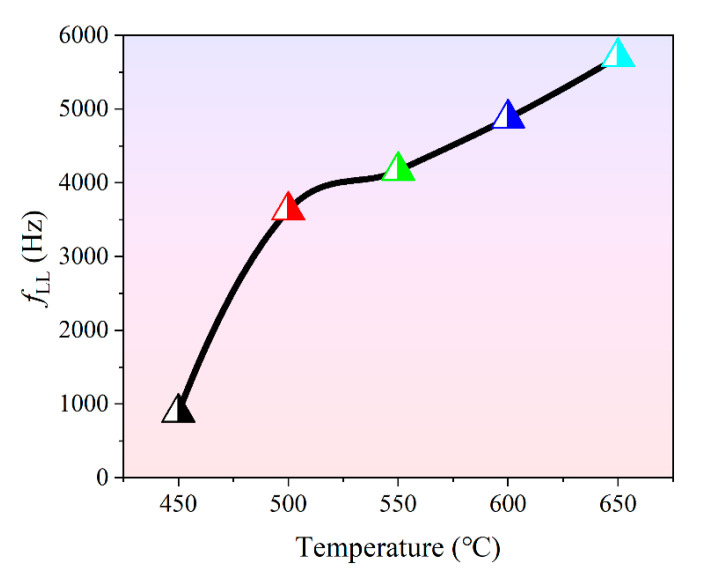
Lower limit frequency (*f_LL_*) of the BGTN−0.2Cr ceramic as a function of temperature.

When poled ferroelectric ceramics are used as piezoelectric elements, over time, the polarization change caused by the applied stress is offset by charge movement caused by internal conduction inside the material. At high frequencies, the charge compensation caused by conductivity can be ignored, because the change rate of charge caused by the applied stress is much faster than the time constant (*RC*). However, at low frequencies, signals from sensors or generators may be significantly attenuated. The minimum useful frequency or lower limit frequency (*f_LL_*) is inversely proportional to the time constant, which can be calculated by Equation (9):

(9) $$f_{LL}=\frac{1}{2\pi RC}=\frac{\sigma }{2\pi \varepsilon^{\prime }}$$

where *σ* is the dc conductivity, which can be deduced from AC fields below 100 Hz, and *ε*′ is the real part of the complex dielectric permittivity. It is well known that the *RC* time constant of BLSF ceramics tends to become very low at high temperature because of the sharp decline of resistivity [40]. In line with Equation (9), the values of *f_LL_* in the temperature range of 450–650 °C were calculated for the BGTN−0.2Cr ceramic and are shown in Figure 4. The values of *f_LL_* showed a sharp rise when the temperature increased from 450 to 500 °C, and then the increase in *f_LL_* with temperature began to slow down when the temperature exceeded 500 °C. In addition to the significant decrease in resistance with temperature, the capacitance of the ferroelectric material is closely related to the temperature. Therefore, the temperature correlation of *f_LL_* combines the effects of capacitance and resistance, which can be considered as a useful quality factor for evaluating the service performance of ferroelectric materials.

### 3.4. Electrical Impedance Spectroscopy of Ceramics

In order to study electrical behavior and distinguish the contribution of grains and grain boundaries to the conductivity of BGTN−0.2Cr ceramic, we analyzed the complex impedance data of BGTN−0.2Cr ceramic, considering both the real (*Z*′) and imaginary (*Z*″) parts. The correlation function relationship can be expressed as follows:

(10) $$Z=Z^{\prime }-jZ^{\prime \prime }$$

(11) $$Z^{\prime }=\frac{R\left(1+{\left(\omega \tau \right)}^{1-\alpha }\cos\left[\left(1-\alpha \right)\frac{\pi }{2}\right]\right)}{1+{\left(\omega \tau \right)}^{2\left(1-\alpha \right)}+2{\left(\omega \tau \right)}^{1-\alpha }\cos\left[\left(1-\alpha \right)\frac{\pi }{2}\right]}$$

(12) $$Z^{\prime \prime }=\frac{R{\left(\omega \tau \right)}^{1-\alpha }\sin\left[\left(1-\alpha \right)\frac{\pi }{2}\right]}{1+{\left(\omega \tau \right)}^{2\left(1-\alpha \right)}+2{\left(\omega \tau \right)}^{1-\alpha }\cos\left[\left(1-\alpha \right)\frac{\pi }{2}\right]}$$

where *τ* = *RC* and *α =* 0~1 is the proportion of the relaxation time distribution.

**Figure 5:**
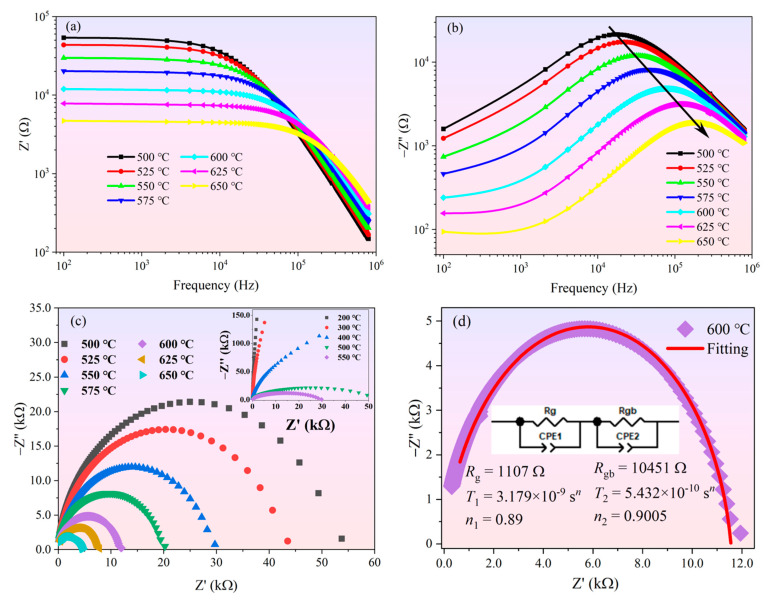
Electrical impedance spectroscopy of the BGTN−0.2Cr ceramic at high temperature: (**a**) *Z*′; (**b**) −*Z*″; (**c**) −*Z*″ vs. *Z*′ (inset shows the impedance curve measured below 550 °C); (**d**) fitting for the Nyquist plot at 600 °C.

It can be seen from Figure 5a that the impedance value had a monotonous reduction as temperature and frequency rose in the low frequency range (≤10 kHz). The reduction of *Z*′ as the temperature rose in the low−frequency part suggests that conductivity increased with temperature. It was also seen that while *Z*′ decreased as frequency increased, after reaching a fixed frequency (≥200 kHz), the value of *Z*′ became higher as the temperature increased and merged when the frequency increased further. This sudden change represents a possibility that the conductivity increased as frequency and temperature rose because of the fixed carriers at low temperatures and the defects at high temperatures. The combination of *Z*′ at all temperatures at high frequencies may be due to the release of space charges, which would have led to a decrease in the resistance of the material. Figure 5b shows the change of *Z″* with frequency at different temperatures. Obviously, as the frequency increased, *Z″* reached a maximum value, which points to the relaxation process in the system. As the temperature increased, the maximum value shifted to higher frequencies. This shows that the relaxation was related to both temperature and frequency. The appropriate temperature activated the particles to cause large motions, and the appropriate frequency caused resonance. When the temperature matched the frequency, the relaxation phenomenon induced was the most obvious (*Z″* maximum). It is well known that in perovskite−type compounds, the short-range motion of oxygen vacancies is a common phenomenon that contributes to high-temperature relaxation [41]. Because of the dispersion of bulk grains, *Z*″ merged at higher frequencies, which signified that the space charge was released. At the same time, the peak value decreased as the temperature increased and tended to become wider. The widening of the peak at higher temperatures indicates the existence of temperature-dependent relaxation. The substances causing relaxation of the material at high temperature may be vacancies or defects [42].

The Nyquist plot (*Z*″ vs. *Z*′) from 200 to 650 °C is shown in Figure 5c. The impedance curve at low temperature (~200 °C) was close to the *y*-axis (imaginary part), which shows the high insulation properties of the ceramic at low temperatures. As the temperature increased, the impedance curve deviated from the *y*-axis and gradually curved toward the *x*-axis (real part) to form a deformed, asymmetric, semicircular arc. The asymmetry from the ideal semicircular arc suggests the possibility of multiple relaxation behaviors in BGTN−0.2Cr ceramic. The temperature continued to rise, and the radius of the deformed semicircular arc gradually decreased, indicating that the resistance of the ceramic gradually decreased as the temperature increased.

In order to judge whether multiple relaxation processes existed in BGTN−0.2Cr ceramic, the complex impedance spectrum was analyzed by z-view simulation software. The results are shown in Figure 5d. In the illustration, a resistor *R_g_* and a constant phase element *Q_g_* (CPE) connected in parallel represent the contribution of the crystal grain, and the other parallel element (*R*_gb_ and *Q_gb_*) represents the contribution of the grain boundary. The total resistance of this equivalent circuit can be expressed as:

(13) $$Z^{*}=\frac{R_{g}}{\left[1+R_{g}T_{1}{\left(i\omega \right)}^{n_{1}}\right]}+\frac{R_{gb}}{\left[1+R_{gb}T_{2}{\left(i\omega \right)}^{n_{2}}\right]}$$

where *R_g_*, *R_gb_*, *T*_1_, and *T*_2_ are the resistance and variables related to the relaxation time distribution from the grain and grain boundaries, respectively; *n* (0~1) is the distribution of relaxation time; and *n* = 1 is the ideal Debye relaxation response. The fitting curve simulated by the Z-View software matched our experimental data well, which confirmed that two kinds of relaxation mechanisms were involved in the BGTN−0.2Cr ceramic, i.e., grain boundaries contributed to the relaxation at low frequency, while grains contributed at high frequency [43]. The results of the simulation analysis showed that the grain boundary resistance (*R_gb_*) was far greater than the bulk grain resistance (*R*_g_) as shown in Figure 5d. This means that at high temperatures, the concentration of oxygen vacancies and captured electronics in the grain boundary was lower. *n*_2_ was greater than *n*_1_, which indicates that the impedance behavior at the grain boundaries was closer to the ideal Debye relaxation model.

**Figure 6:**
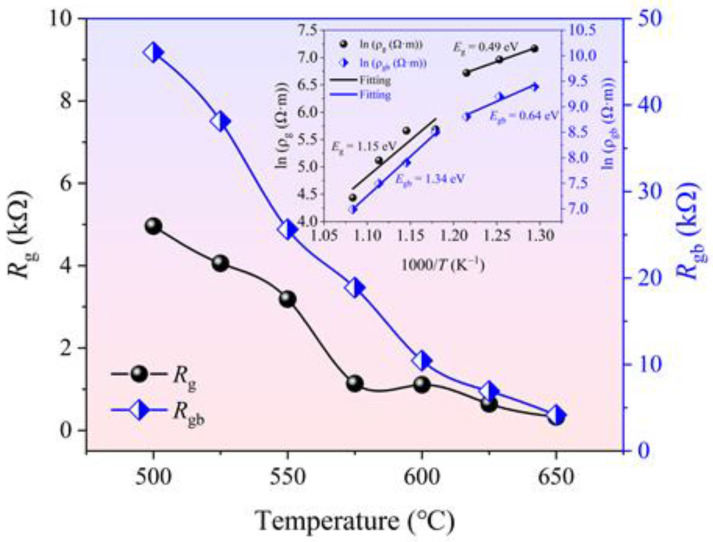
Temperature dependence of *R_g_* and *R_gb_* of the BGTN−0.2Cr ceramic (inset shows the Arrhenius fitting of the plots of resistivity vs. temperature).

The temperature dependence of *R_g_* and *R_gb_* of the BGTN−0.2Cr ceramic is displayed in Figure 6. The rate at which the grain’s resistance decreased as the temperature rose was slower than that of the grain boundary. The calculation of resistivity and its temperature dependence can be described by the following formulas:

(14) $$\rho =\frac{RS}{L}$$

(15) $$\rho =\rho_{0}\exp\left(E_{\mathrm{con}}/kT\right)$$

where *E_c__on_* is the activation energy for conduction, *ρ*_0_ is the pre-exponential factor, and *k* is the Boltzmann constant. As shown in Figure 6, as the temperature rose, *E*_g_ increased from 0.49 to 1.15 eV, while *E_gb_* increases from 0.64 to 1.34 eV. Such a significant increase in the activation energy confirmed that the charge carriers responsible for the electrical conduction process at high temperature changed from the primary−ionized oxygen vacancies to the secondary-ionized ones. No matter what kind of charge carriers dominated the electrical conduction, *E_g_* was always lower than *E_gb_*, which implies that the carrier concentration or migration speed of the grains was higher than that of the grain boundaries.

### 3.5. Electrical Modulus Spectroscopy of Ceramics

The impedance spectrum data emphasized only the maximum resistance element of microscopic components. In order to better understand the relaxation behaviors in BGTN−0.2Cr ceramic, electrical modulus analysis and impedance analysis complemented each other. The modulus spectrum handles the minimum capacitive element of the microscopic components and can suppress electrode interface effects [44,45]. Physically, the electrical modulus corresponds to the relaxation of the electric field in the material when the electric displacement remains constant. Therefore, the electrical modulus represents the real dielectric relaxation process, which can be expressed as:

(16) $$M=M^{\prime }+j\;M^{\prime \prime }=j\omega C_{0}Z$$

(17) $$M^{\prime }=\omega C_{0}Z^{\prime \prime }$$

(18) $$M^{\prime \prime }=j\omega C_{0}Z\prime$$

where *C*_0_ is the capacitance of free space given by *C*_0_ = *ε*_0_*A/d* [46].

**Figure 7:**
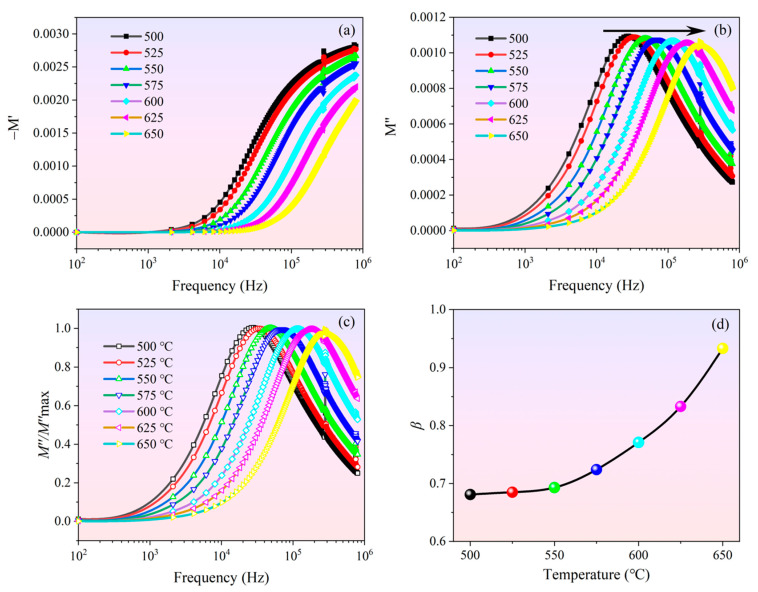
Electrical modulus spectroscopy of the BGTN−0.2Cr ceramic at high temperature: (**a**) −*M*′; (**b**) *M*″; (**c**) *M*″/*M*″*_max_*; (**d**) temperature dependence of *β*.

**Figure 8:**
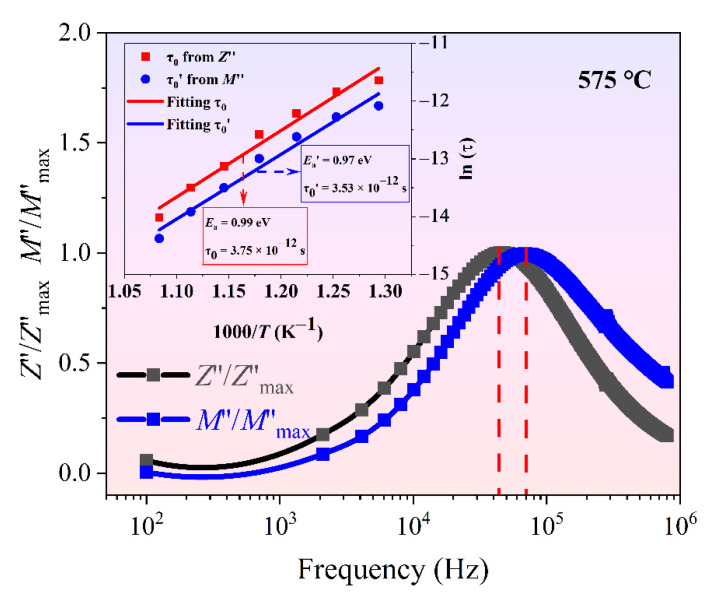
Comparison between *Z*″/*Z*″*_max_* peak and *M*″/*M*″*_max_* peak for the BGTN−0.2Cr ceramic (inset shows the Arrhenius fitting of the plots of relaxation time vs. temperature).

In Figure 7, the electrical modulus spectroscopy of the BGTN−0.2Cr ceramic at high temperature is shown as a function of the frequency with temperature changing. It is obvious from Figure 7a that every temperature showed an identical trend—as frequency rose, *M*′ values increased, gradually slowing (*M*′ gradually in a fixed value at higher frequencies). *M*′ showed asymmetry because of the tensile index characteristics of the relaxation time of materials. The monotonic dispersion in the low-frequency area may be due to the short-range jump of the carriers. This result may be related to the lack of recovery power of the charge carrier migration under the control of the polarization electric field [47]. On the other hand, *M*″ increased as frequency rose and reached a peak of relaxation because of the bulk grain and grain boundary behaviors. In physics, the electrical modulus peak can determine the area that the charge carrier can be migrated long distance. The asymmetrical and wide *M*″ peaks imply that the nonexponential behaviors of the grain and grain boundary relaxation deviated from Debye-type relaxation. The behavior indicates that ion migration occurred by jumping accompanying the corresponding time-dependent mobility of other nearby charge carriers [48]. These relaxation peaks moved towards higher frequencies as the temperature increased. With the equation ωτ = 1, we can obtain the relaxation time ${\tau_{0}}^{\prime }$ related to the electrical modulus and perform Arrhenius fitting to ${\tau_{0}}^{\prime }$. Comparing the activation energies obtained from the impedance and modulus spectra (Figure 8), we found that the two processes had similar activation energies and pre-exponential factors. This indicates that the two had a common relaxation mechanism—both were dominated by similar carriers. The grain boundaries contributed to low-frequency relaxation, and the grains contributed to high-frequency relaxation.

The *M*″ curves at different temperatures were normalized to a master curve with the peak position (*f_max_*) and the peak height (*M*″*_max_*) to research the relaxation process (Figure 7c). The shape of this peak was asymmetrical and wider than Debye-type relaxation. This phenomenon is well described by the Bergman formula [49]:

(19) $$M^{\prime \prime }\left(\omega \right)=\frac{{M^{\prime \prime }}_{max}}{1-\beta +\frac{\beta }{1+\beta }{\left[\beta \left(\omega_{max}/\omega \right)+\left(\omega /\omega_{max}\right)\right]}^{\beta }}$$

where *M*″*_max_* is the maximum value of *M*″, *ω_max_* is the angular frequency corresponding to *M*″*_max_*, and *β* indicates the extent of deviation from the ideal Debye model—the smaller the value of *β*, the greater the deviation from Debye−type relaxation (*β* = 1, see [48]). *β* increased as temperature rose (Figure 7d), which proves that the relaxation behavior of the BGTN−0.2Cr ceramic grew closer to Debye−type relaxation at higher temperature.

In Figure 8, the frequency dependence of *Z*″/*Z*″*_max_* and *M*″/*M*″*_max_* at 575 °C is shown. It was discovered that the impedance and electrical modulus each showed only one peak of relaxation behaviors. Because of the different focuses of the impedance and modulus, the impedance spectrum with the more resistive grain-boundary component displayed only a single peak, while the single peak of *M*″/*M*″*_max_* may be due to the contribution of the grains. The *Z*″/*Z*″*_max_* peak appeared at a lower frequency, which suggests that the grain boundary gathered a large amount of oxygen vacancies and space charges. The *M*″/*M*″*_max_* peak appeared at a higher frequency, which suggests that the grain has a carrier concentration or migration speed below the grain boundary. *E_a_* dominated by *Z* was slightly larger than *E_a_*’ dominated by *M*, which once again proves that the activation energy of the grain boundary was slightly larger than that of the grain, as shown in Figure 6.

### 3.6. Electromechanical Resonance Spectroscopy of Ceramics

**Figure 9:**
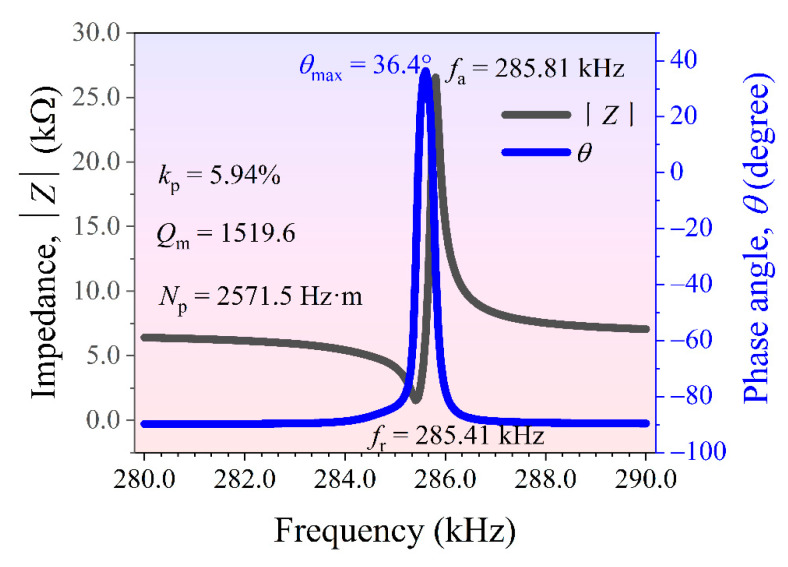
Electromechanical resonance spectroscopy of the BGTN−0.2Cr ceramic at room temperature.

Figure 9 shows the electromechanical resonance spectroscopy results for the BTGN−0.2Cr ceramic at room temperature. This measured the frequency dependence of impedance |*Z*| and the phase angle *θ* of the piezoceramic as a resonator. The paired peak of resonance-antiresonance around 285.5 kHz showed that the sample was in the radial-extensional vibration mode. In an ideal poling state, piezoelectric materials should exhibit an impedance phase angle *θ* approaching 90° in the frequency range between the resonance (*f_r_*) and antiresonance frequencies (*f_a_*). As can be seen, the maximum phase angle *θ_max_* was only 36.4°, indicating the insufficient poling of the sample. Since BTN has a high coercive electric field, as well as a low resistivity, a complete domain switching is difficultly achieved by the poling electric field. Things that give satisfaction is that the *d*_33_ value of the BTGN−0.2Cr ceramic has reached 18 pC/N, which provides it with a large competitiveness used as sensing materials for high−temperature piezoelectric sensors.

## 4. Conclusions

An Aurivillius phase ceramic with a formula of Bi_2.8_Gd_0.2_TiNbO_9_ + 0.2 wt% Cr_2_O_3_ (abbreviated as BGTN−0.2Cr) was prepared by the conventional solid−state reaction route. Both the microstructures and electrical conduction behaviors of the BGTN−0.2Cr ceramic were studied. The BGTN−0.2Cr ceramic was crystallized in a pure Bi_3_TiNbO_9_ phase and composed of plate-like grains. Its ac conduction behavior could be explained by the Funke’s jumping relaxation model. The maximum barrier height *W_M_*, hopping conduction activation energy *E_p_*, and dc conduction activation energy *E_c_* were determined to have values of 0.63 eV, 1.09 eV, and 0.73 eV, respectively. Impedance analysis in combination with modulus calculation revealed that grains provided a larger contribution to the conductance at high temperature and that *β* grew larger as temperature rose. The values of the activation energy (~1 eV) calculated from both *Z″* and *M*″ peaks suggested the electrical relaxation process to be dominated by the thermal activation of oxygen vacancies as defect charge carriers. Moreover, the BGTN−0.2Cr ceramic had a high *d*_33_ of 18 pC/N as well as a high *σ*_*dc*_ of 1.52 × 10^5^ Ω cm (600 °C). Such excellent electrical properties made it a competitive candidate for high−temperature piezoelectric materials.

## Data Availability

The data presented in this study are available on request from the corresponding author.
